# Myocardial Performance after Successful Liver Transplantation

**Published:** 2016-05-01

**Authors:** H. Amoozgar, R. Ermis, N. Honar, S. A. Malek-Hosseini

**Affiliations:** 1 *Neonatology and Cardiac Research Center, Shiraz, Iran*; 2 *Department of Pediatrics, Shiraz University of Medical Sciences, Shiraz, Iran*; 3 *Pediatric Gastroenterology and Hepatology Ward, Shiraz University of Medical Sciences, Shiraz, Iran*; 4 *Transplant Research Center, Nemazi Hospital, Shiraz University of Medical Sciences, Shiraz, Iran*

**Keywords:** Myocardium, Karnofsky performance Status, Liver transplantation, Child, Tei index, Liver cirrhosis

## Abstract

**Background:**

Myocardial performance index (MPI) or Tei index is a Doppler-derived index of combined systolic and diastolic myocardial function, calculated as the sum of isovolumetric relaxation time (IRT) and isovolumetric contraction time (ICT) divided by the ejection time (ET).

**Objective:**

To evaluate the right and left ventricular systolic and diastolic function using MPI in children before and after liver transplantation.

**Methods:**

A cross-sectional study was conducted on 30 children with liver cirrhosis before liver transplantation, 30 age-matched comparison group at least 6 months after liver transplantation, and 30 aged-matched children without history of heart disease in Nemazi Liver Transplant Center, Shiraz, Iran, from April 2012 to April 2014. Echocardiographic evaluation was carried out with a GE Vivid 3 echocardiographic machine, using a 3-MHz probe with tissue Doppler imaging (TDI) software using conventional and TDI method.

**Results:**

The mean±SD left ventricle Tei index in patients was 0.33±0.02 before liver transplantation, 0.34±0.02 after liver transplantation, and 0.33±003 in the comparison group (p=0.36). The mean±SD right ventricular Tei index was 0.35±0.04 in patients before transplantation, 0.36±0.46 after liver transplantation, and 0.28±0.04 in the comparison group (p<0.001). In addition, when TDI was used, the mean±SD left ventricular Tei index was 0.39±0.50 in patients before transplantation, 0.37±0.42 after liver transplantation, and 0.38±006 in the comparison group (p=0.32). The tissue Doppler-derived Tei index for the right ventricle was 0.37±0.04 in patients before transplantation, 0.37±0.04 after liver transplantation, and 0.33±0.05 in the comparison group (p=0.031). The left ventricular Doppler-derived Tei index had a significant (p=0.03) correlation with Child-Turcotte-Pugh (CTP) score (r=0.57).

**Conclusion:**

Left ventricular MPI with Doppler echocardiography was correlated with CTP score. Right ventricular MPI was significantly increased in patients with cirrhosis and did not improve 6 months after transplantation.

## INTRODUCTION

Cirrhosis is a potential end-result of any progressive liver diseases, defined histologically by the presence of bands of fibrous tissue that link the central and portal areas and form parenchymal nodules. It can be post-hepatitis (after acute or chronic hepatitis) or post-necrotic (after toxic injury), or it can caused by intra-hepatic or extra-hepatic obstruction of bile flow [1]. In children, cirrhosis is mostly caused by chronic cholestasis, which is started early in life, inborn error of metabolism and chronic hepatitis [2].

Cirrhosis is a condition that has a variety of clinical manifestations and complications, some of which can be life-threatening such as cardiovascular complications, portal hypertension, hepatorenal syndrome, hepatic encephalopathy, and porto-pulmonary hypertension [3].

Cardiovascular complications of cirrhosis present with (a) reduction in afterload and then in preload caused by the central, splanchnic and peripheral circulation aberrancy and hemodynamic alterations triggered by humoral and nervous dysregulation; (b) cirrhotic cardiomyopathy characterized by presence of increased baseline cardiac output, systolic and diastolic dysfunction and electrophysiological abnormalities; and (c) porto-pulmonary hypertension [4, 5].

Liver transplantation should be considered for patients with decompensated cirrhosis [3]. The most common indication for pediatric liver transplantation is biliary atresia [6-8]. Nowadays, a Doppler-derived index referred to as the “Tei index” or “myocardial performance index” (MPI) that incorporates systolic and diastolic time intervals, has been developed to estimate the global cardiac function [9].

MPI was firstly introduced by Chuwa Tei and colleagues in 1995, as a simple and reproducible Doppler index of combined systolic and diastolic myocardial performance in patients with primary myocardial systolic dysfunction, calculated as the sum of the isovolumetric relaxation time (IRT) and isovolumetric contraction time (ICT) divided by the ejection time (ET) [10].

The objective of this study was to evaluate the right and left ventricular systolic and diastolic function, using MPI, in children before and after liver transplantation.

## PATIENTS AND METHODS

In this cross-sectional study 30 children with liver cirrhosis before liver transplantation, 30 age-matched comparison group at least 6 months after liver transplantation, and 30 aged-matched children without a history of heart disease were investigated in Nemazi Liver Transplant Center, Shiraz, Iran, from April 2012 to April 2014. The patients’ demographic data including weight, age, and sex were recorded. PELD-MELD score and the Child-Turcotte-Pugh (CTP) scores were calculated to evaluate liver disease severity. [12-14]. Blood specimens were collected and tested for albumin (Alb), globulin (GLB), aspartate aminotransferase (AST), alanine aminotransferase (ALT), alkaline phosphatase (AlkPh), total bilirubin (TB), direct bilirubin (DB), sodium (Na), potassium (K), calcium (Ca), phosphorus (P), blood urea nitrogen (BUN), creatinine (Cr). The inclusion criteria included age less than 18 years, having end-stage liver disease, being a candidate for liver transplantation. Patients with at least six months after successful liver transplantation were included as comparison group. The patients with known congenital heart disease, renal disease and acute heart failure were excluded from the study. 

Study Protocol

The study was approved by the research ethics committee of the university, and written informed consents were obtained from patients’ guardians. Age-matched pediatric patients who had no heart disease, underwent the same echocardiographic studies as the comparison group.

Echocardiographic Examination

Echocardiography was performed with a GE Vivid 3 echocardiographic machine, using a 3-MHz probe with tissue Doppler imaging (TDI) software. Tei index was obtained by conventional and TDI method. For Doppler interrogation, we measured three consecutive beats, averaging the values, and assessing the interval from the cessation to the onset of mitral or tricuspid inflow, equal to the sum of the isovolumetric contraction time (ICT), ejection time, and isovolumetric relaxation time (IRT), designated as ‘a’ in the equation shown below. The duration of ventricular ejection from the onset to the end of aortic or pulmonary ejection was designated as ‘b’ in the equation. The Doppler-derived Tei index, combining systolic and diastolic function of either ventricle, was then calculated as (a–b)/b.

TDI was obtained with the sample volume placed on the lateral corner of the mitral annulus and, subsequently, on the medial (or septal) corner from the apical four-chamber view. In each region, systolic (S) wave, early diastolic (Ea), and late diastolic (Aa) velocities and ejection time (ET) were recorded. Also, the ICT and the IRT were measured from the end of the mitral annular velocity pattern to the onset of the S wave and from the end of the S wave to the onset of the mitral annular velocity pattern, respectively. The TDI-derived Tei index (TDI-Tei), sum of ICT, and IRT divided by ET, was determined for the lateral mitral annulus. Time interval measurements were obtained from three consecutive beats, and then the data were averaged.

Statistical Analysis

Statistical analyses were performed by SPSS^®^ for Windows^®^ ver 16 using one-way ANOVA, *Student’s t* test, and Pearson’s correlation analysis. A p value <0.05 was considered statistically significant.

## RESULT

The median age of the three studied groups was 5.0 (range: 0.6–18) years. Sixteen (53%) patients were male and 24 (46%) were female. Laboratory data before and after liver transplantation are summarized in Table 1. Total and direct bilirubin were significantly lower in the post-transplantation group in comparison to the pre-transplantation patients (p=0.009 and p=0.039, respectively). Other blood test results were not significantly different. The mean±SD CTP score was 7.41±2.35 in patients before liver transplantation, and 7.72±2.21 after transplantation (p=0.644). The mean PELD-MELD was not statistically different before and after transplantation (p=0.598).

**Table 1:** Distribution of pre- and post-transplantation patients according to weight, blood tests and liver grading score. Figures are mean±SD.

Parameter	Pre-transplantation	Post-transplantation	p value
Weight (kg)	22.8±17.5	31.9±18.6	0.037
Alb (g/dL)	3.94±0.80	3.82±0.73	0.622
Glb (g/dL)	3.69±1.12	3.34±1.01	0.330
AST (U/L)	148±137	131±171	0.709
ALT (U/L)	73.4±58.6	123±197	0.195
AlkPh (U/L)	973±615	908±846	0.759
TB (mg/dL)	10.7±13.1	2.15±2.26	0.009
DB (mg/dL)	3.08±4.35	0.85±1.10	0.039
Na (mEq/L)	139±4.45	138±5.65	0.691
K (mEq/L)	4.21±0.39	4.32±0.58	0.411
Ca (mEq/L)	8.80±1.36	8.21±1.88	0.264
P (mg/dL)	5.78±1.50	6.24±1.36	0.311
BUN (mg/dL)	10.9±6.9	14.33±7.94	0.131
Cr (mg/dL)	0.56±0.21	0.52±0.25	0.505
CTP score	7.41±2.35	7.72±2.21	0.644
PELD-MELD	18.0±9.61	16.1±9.70	0.598

Alb: Albumin; Glb: Globulin; AST: Aspartate aminotransferase; ALT: Alanine aminotransferase; AlkPh: Alkaline phosphatase; TB: Total bilirubin; DB: Direct bilirubin; Na: Sodium; K: Potassium; Ca: Calcium; P: Phosphorus; BUN: Blood urea nitrogen; Cr: Creatinine; CTP score: Child-Turcotte-Pugh

M-mode echocardiographic findings of patients before liver transplantation were not significantly different from those of the comparison group (Table 2). In all patients before and after liver transplantation, EF was within the normal range (55%–75%) or higher. Other parameters showed no significant differences.

**Table 2:** M-mode echocardiographic parameters in pre- and post-transplantation patients. Figures are mean±SD

Parameter	Pre-transplantation	post-transplantation	p value
EF (%)	74.7±8.11	70.5±10.4	0.142
EDD (mm)	58.7±27.2	59.9±24.5	0.919
ESD (mm)	18.4±19	24.4±25.1	0.515
IVSS (mm)	97.4±28.1	106±31.04	0.300
IVSD (mm)	82.1±24.3	87.6±16.3	0.401
LVPWS (mm)	79.2±22.2	90.7±22.4	0.096
LVPWD (mm)	68.3±14.7	66.5±19.3	0.727

EF: Ejection fraction; EDD: End-diastolic diameter; ESD: End-systolic diameter; IVSS: Inter-ventricular septum in systole; IVSD: Inter-ventricular septum in diastole; LVPWS: Left-ventricular posterior wall in systole; LVPWD: Left-ventricular posterior wall in diastole

Doppler echocardiographic parameters are shown in Table 3. Pulmonary artery acceleration time in patients was significantly (p=0.002) different from that in the comparison group. Other measured parameters were not significant different. The left ventricular mean±SD Tei index was 0.33±0.02 in patients before transplantation, 0.34±0.02 after liver transplantation, and 0.33±003 in the comparison group (p=0.36). The right ventricular mean±SD Tei index was 0.35±0.04 in patients before transplantation, 0.36±0.46 after liver transplantation, and 0.28±0.04 in the comparison group (p<0.001).

**Table 3:** Doppler echocardiographic parameters of pre- and post-transplantation patients. Figures are mean±SD

Parameter	Pre-transplantation	Post-transplantation	p value
PAT (ms)	125±39.2	247±13.8	0.002
Em (cm/s)	108±20.4	104±14.3	0.571
Am (cm/s)	62.3±24.0	63.7±11.7	0.826
Em/AM ratio	2.02±1.19	1.69±0.36	0.256
Et (cm/s)	78.4±18.9	78.8±18.9	0.941
At (cm/s)	54.2±20.3	56.8±17.3	0.649
Et/At ratio	1.62±0.69	1.46±0.37	0.401
Et/Am ratio	2.02±1.19	1.69±0.36	0.256
LVMPI	0.33±0.02	0.34±0.02	0.320
RVMPI	0.35±0.04	0.36±0.46	0.313

PAT: Pulmonary artery acceleration time; Em: Early diastolic velocity of mitral; Am: Late diastolic velocity of mitral; Et: Early diastolic velocity of tricuspid; At: Late diastolic velocity of tricuspid; LVMPI: Left ventricular myocardial performance index; RVMPI: Right ventricular myocardial performance index

Tissue Doppler echocardiographic parameters are shown in Table 4. Early diastolic velocity of the septum showed a statistically significant value (p=0.032) but other findings did not. The mean of the left ventricular Tissue Doppler derived Tei index was 0.39±0.50 in patients before transplant, 0.37±0.42 after liver transplant, and 0.38±006 in the controls, which was not significantly different (p=0.32). The mean of the right ventricular Tissue Doppler derived Tei index was 0.37±0.04 in patients before transplant, 0.37±0.04 after liver transplant, and 0.33 ± 0.05 in the controls, which was statistically different (p=0.031). 

**Table 4:** Tissue doppler echocardiographic parameters of pre- and post-transplantation patients. Figures are mean±SD

Parameter	Pre-transplantation	Post-transplantation	p value
Sam (cm/s)	9.59±1.90	9.33±2.00	0.666
Eam (cm/s)	17.7±5.31	17.4±3.76	0.809
Aam (cm/s)	9.59±3.29	8.63±2.55	0.318
Em/EaM ratio	6.64±2.39	6.33±1.84	0.646
Ss (cm/s)	8.52±1.37	8.67±1.28	0.713
Eas (cm/s)	14.3±3.02	12.6±1.91	0.032
Aas (cm/s)	9.0±2.46	9.06±2.79	0.939
St (cm/s)	13.8±3.06	13.3±2.11	0.535
Eat (cm/s)	17.1±5.20	16.1±4.24	0.509
Aat (cm/s)	13.9±4.59	13.3±4.92	0.694
LVMPI	0.39±0.05	0.37±0.04	0.281
RVMPI	0.37±0.04	0.36±0.04	0.422

Sam: Systolic velocity of mitral; Eam: Early diastolic velocity of mitral; Aam: Late diastolic velocity of mitral; Eas: Early diastolic velocity of septum; Aas: Late diastolic velocity of septum; Eat: Early diastolic velocity of tricuspid; Aat: Late diastolic velocity of septum; LVMPI: Left ventricular myocardial performance index; RVMPI: Right ventricular myocardial performance index

We also evaluated the correlation between echocardiographic parameters, demographic data and blood tests. The left ventricular Doppler-derived Tei index had a significant (p=0.03) correlation with CTP score (r=0.57). The PELD-MELD score did not have any significant correlation with LVMPI measured by Doppler echocardiography.

## DISCUSSION

We studied cardiac systolic and diastolic function before and after liver transplantation in children with cirrhosis. We found that the echocardiographic parameters of children with cirrhosis measured before and after transplant did not have significantly different. This means that the right and left ventricular systolic and diastolic function did not improve six months after liver transplantation. Furthermore, the left ventricular MPI echocardiography showed a correlation with CTP score level. In addition, the right ventricular MPI in patients before and after transplantation was significantly higher than the that in the comparison group. 

Cirrhosis-associated cardiomyopathy is defined as impaired contractile responsiveness to stress or altered diastolic function with electrophysiologic abnormalities in the absence of other known cardiac disease. This syndrome is seen in approximately 40%–50% of adult patients with cirrhosis [11]. The incidence of this syndrome in pediatric patients is not well known. The clinical manifestations of this syndrome correlate with the severity of cirrhosis in adults [12]. 

Clinical characterization of cirrhosis-associated cardiomyopathy includes systolic dysfunction, diastolic dysfunction, pulmonary hypertension, and electrophysiological dysfunction. The high resting cardiac output and lower filling pressures in patients with cirrhosis are partially explained by low systemic vascular resistance and increased arterial compliance [13]. 

Diastolic dysfunction is a prominent feature of CAC. This describes impairment of ventricular filling as a result of alterations in the receptive ventricular properties. The underlying mechanism of diastolic dysfunction in cirrhosis is increased myocardial wall stiffness, most likely due to myocardial hypertrophy, fibrosis, and sub-endothelial edema [13], resulting in high filling pressure of the left ventricle and atrium that ultimately increases the risk of pulmonary edema because of the backward failure.

Hepatic dysfunction is categorized by CTP score; we can see more increase in cardiac output in response to exercise in patients with mild liver damage compared to patients with moderate to severe liver damage [14].

Hepatic cirrhosis is associated with systemic circulatory and cardiac changes that are hyperdynamic circulation with increased cardiac output and heart rate and reduced systemic vascular resistance over time due to splanchnic arterial vasodilation [15]. Patients with hepatic cirrhosis also have defects in both systolic and diastolic dysfunction that only become obvious with physiologic stress such as liver transplantation by causing a sudden reduction in the cardiac preload that results in decreased cardiac output [16, 17]. However, it has been shown that liver transplantation normalizes hepatic metabolism and improves circulatory alterations including the hyperdynamic state [16, 15]. Some studies have assessed the incidence of cardiovascular events in the post-liver transplantation period. Torregrosa, *et al* [18], reported that cardiac abnormalities in end-stage liver disease are completely reversible between 6 and 12 months after liver transplantation. Yang, *et al* [16], also reported that liver transplantation improves most of the functions of the heart. However, Acosta, *et al* [19], reported that cirrhotic patients undergoing orthotopic liver transplantation present with pre-operative normal cardiac function progress with significant diastolic dysfunction after transplantation, perhaps as a result of immunosuppressive treatment. In addition, Dec, *et al* [20], reported that cardiovascular complications happened in over 70% of post-transplantation patients during the first six months following liver transplantation. 

Pulmonary acceleration time is increased in post-transplantation period that could be an indirect evidence of decreased pulmonary pressure in post-transplant recipients. Increased left ventricular posterior wall diameter and inter-ventricular wall thickening in pre- and post-liver transplantation patients may represent positive response to increase in the end-diastolic volume. 

In conclusion, left ventricular MPI measured with Doppler echocardiography is correlated with CTP score level. Right ventricular MPI is significantly increased in patients with cirrhosis and does not improve six months after transplantation.

## References

[1] Lynelle MB, William FB, Robert MK (2011). Manifestations of liver disease. Nelson Textbook of Pediatrics.

[2] Bernard O (1997). Cirrhosis in children. Rev Prat.

[3] Bruce RB, Dan LL (2012). Cirrhosis and its complications. Harrison’s Principles of Internal Medicine.

[4] Møller S, Henriksen JH (2009). Cardiovascular complications of cirrhosis. Postgrad Med J.

[5] Ripoll C, Yotti R, Bermejo J, Banares R (2011). The heart in liver transplantation. J of Hepatology.

[6] Dehghani SM, Gholami S, Bahador A (2007). Morbidity and mortality of children with chronic liver diseases who were listed for liver transplantation in Iran. Pediatr Transplant.

[7] Debray D, Bernard O, Gauthier F (2009). [Pediatric liver transplantation]. Presse Med.

[8] Dalgic A, OzcayF, ArslanG (2005). Living-related liver transplantation in pediatric patients. Transplant Proc.

[9] Pellett AA, Tolar WG, Merwin DG, Kerut EK (2004). The Tei index: methodology and disease state values. Echocardiography.

[10] Tei C, Ling LH, Hodge DO (1995). New index of combined systolic and diastolic myocardial performance: a simple and reproducible measure of cardiac function – a study in normal and dilated cardiomyopathy. J Cardiol.

[11] Møller S, HenriksenJH (2010). Cirrhotic cardiomyopathy. J Hepatol.

[12] Liu H, Gaskari SA, Lee SS (2006). Cardiac and vascular changes in cirrhosis: pathogenic mechanisms. World J Gastroenterol.

[13] Møller S, Henriksen JH (2001). Cardiovascular dysfunction in cirrhosis Pathophysiological evidence of a cirrhotic cardiomyopathy. Scand J Gastroenterol.

[14] Grose RD, Nolan J, Dillon JF (1995). Exercise induced left ventricular dysfunction in alcoholic and non alcoholic cirrhosis. J Hepatol.

[15] Møller S D, Hove J, Dixen U, Bendtsen F (2013). New insights into cirrhotic cardiomyopathy. Int J Cardiol.

[16] Yang YY, Lin HC (2012). The heart: Pathophysiology and clinical implications of cirrhotic cardiomyopathy. J Chin Med Assoc.

[17] Mandell MS, Tsou MY (2008). Cardiovascular dysfunction in patients with end-stage liver disease. J Chin Med Assoc.

[18] Torregrosa M, Aguade S, Dos L (2005). Cardiac alterations in cirrhosis: reversibility after liver transplantation. J Hepatol.

[19] Acosta F, De La Morena G, Villegas M (1999). Evaluation of cardiac function before and after liver transplantation. Transplant Proc.

[20] Dec GW, Kondo N, Farrell ML (1995). Cardiovascular complications following liver transplantation. Clin Transplant.

